# An Ethanol Extract of *Perilla frutescens* Leaves Suppresses Adrenergic Agonist-Induced Metastatic Ability of Cancer Cells by Inhibiting Src-Mediated EMT

**DOI:** 10.3390/molecules28083414

**Published:** 2023-04-12

**Authors:** Jae-Hoon Jeong, Hyun-Ji Park, Gyoo-Yong Chi, Yung-Hyun Choi, Shin-Hyung Park

**Affiliations:** 1 Department of Pathology, College of Korean Medicine, Dong-eui University, Busan 47227, Republic of Korea; 15224@deu.ac.kr (J.-H.J.); 14554@deu.ac.kr (H.-J.P.); cgyu@deu.ac.kr (G.-Y.C.); 2 Department of Biochemistry, College of Korean Medicine, Dong-eui University, Busan 47227, Republic of Korea

**Keywords:** adrenergic signaling pathway, cancer, invasion, migration, EMT, Src

## Abstract

Previous studies have indicated that the adrenergic receptor signaling pathway plays a fundamental role in chronic stress-induced cancer metastasis. In this study, we investigated whether an ethanol extract of *Perilla frutescens* leaves (EPF) traditionally used to treat stress-related symptoms by moving Qi could regulate the adrenergic agonist-induced metastatic ability of cancer cells. Our results show that adrenergic agonists including norepinephrine (NE), epinephrine (E), and isoproterenol (ISO) increased migration and invasion of MDA-MB-231 human breast cancer cells and Hep3B human hepatocellular carcinoma cells. However, such increases were completely abrogated by EPF treatment. E/NE induced downregulation of E-cadherin and upregulation of N-cadherin, Snail, and Slug. Such effects were clearly reversed by pretreatment with EPF, suggesting that the antimetastatic activity of EPF could be related to epithelial–mesenchymal transition (EMT) regulation. EPF suppressed E/NE-stimulated Src phosphorylation. Inhibition of Src kinase activity with dasatinib completely suppressed the E/NE-induced EMT process. Transfecting MDA-MB-231 cells with constitutively activated Src (SrcY527F) diminished the antimigration effect of EPF. Taken together, our results demonstrate that EPF can suppress the adrenergic agonist-promoted metastatic ability of cancer cells by inhibiting Src-mediated EMT. This study provides basic evidence supporting the probable use of EPF to prevent metastasis in cancer patients, especially those under chronic stress.

## 1. Introduction

Associations of chronic psychological stress with initiation and progression of cancer have been continuously studied in recent past decades. Clinical studies have reported that cancer patients under stress have a greater risk of recurrence and higher mortality than those without such stress [1,2]. Preclinical studies have also shown that chronic stress can accelerate cancer growth, angiogenesis, and metastasis in mouse models [3,4,5]. On the other hand, psychosocial intervention can improve the survival rate and decrease recurrence in cancer patients [6,7].

Under stressful conditions, the sympathetic nervous system is activated, which, in turn, stimulates the release of stress hormones including epinephrine (E) and norepinephrine (NE) from the adrenal medulla or from sympathetic nerve fibers [8]. These catecholamines can bind to β-adrenergic receptors (ARs) and activate protein kinase A (PKA), which phosphorylates multiple target proteins such as activating transcription factor (ATF), cAMP response element binding protein (CREB), and Src to promote cancer cell proliferation, migration, and cellular resistance to apoptosis [9]. As β-ARs are present in a variety of cancer cells, as well as stromal cells, within the tumor microenvironment, regulating the β-AR signaling pathway has been considered a promising strategy for the management of cancer [10,11]. It has been shown that consumption of β blockers can improve relapse-free survival, decrease metastasis in breast cancer, and reduce the cancer-specific mortality rate in pancreatic cancer [12,13,14]. Long-term use of β blockers can also decrease cancer risk and improve the overall survival of liver cancer patients [15,16]. These observations have expanded the application of β blockers from classical cardiovascular diseases to cancer therapy.

Cancer metastasis is a major hurdle for cancer therapy, as it is closely associated with poor prognosis and low quality of life in cancer patients [17]. As a key mediator of this event, the epithelial–mesenchymal transition (EMT) program enables cancer cells to acquire apolar, motile, and mesenchymal-like phenotypes and invade local tissues or blood vessels to metastasize to distal organs [18]. Interestingly, activation of β-ARs is known to promote metastasis by stimulating EMT, suggesting that blocking the EMT process could be a probable strategy to handle chronic stress-related metastasis [9].

**Figure 1:**
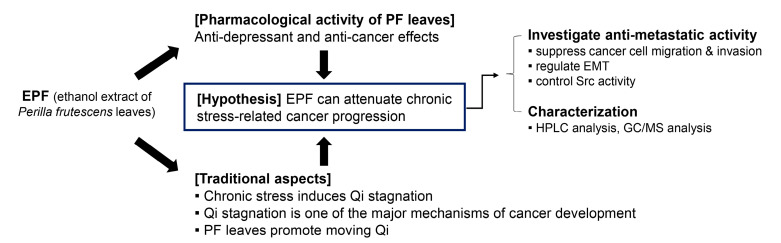
The hypothesis and methodology of this study.

*Perilla frutescens* (PF) leaves have traditionally been prescribed in East Asian countries to treat respiratory diseases and gastrointestinal disorders such as asthma, colds, flu, vomiting, and abdominal pain. Other important indications for the use of PF leaves include psychological disorders such as depression and anxiety based on a traditional theory that PF leaves can promote circulation of Qi [19]. Modern research studies have reported a variety of pharmacological activities of PF leaves, including antioxidant, antibacterial, antiallergic, antidepressant, anti-inflammatory, and anticancer effects [19]. Crude extracts or several constituents of PF leaves exhibit anticancer properties by inhibiting carcinogenesis and inducing apoptosis, cell cycle arrest, and cellular senescence in cancer cells [19,20,21]. However, effects of PF leaves on chronic stress-induced cancer progression have not been reported yet. According to the theory of traditional Oriental medicine, chronic stress can induce Qi stagnation, which is one of the major mechanisms of cancer development [22,23,24]. Based on the traditional role of PF leaves in moving Qi and the anticancer properties of PF leaves, we hypothesized that PF leaves could attenuate chronic stress-related cancer progression. In this study, we investigated whether an ethanol extract of PF leaves (EPF) could inhibit the adrenergic agonist-induced metastatic ability of cancer cells and explored the underlying mechanism (Figure 1).

## 2. Results

### 2.1. High-Performance Liquid Chromatography (HPLC) Analysis of Marker Constituents in EPF

**Figure 2:**
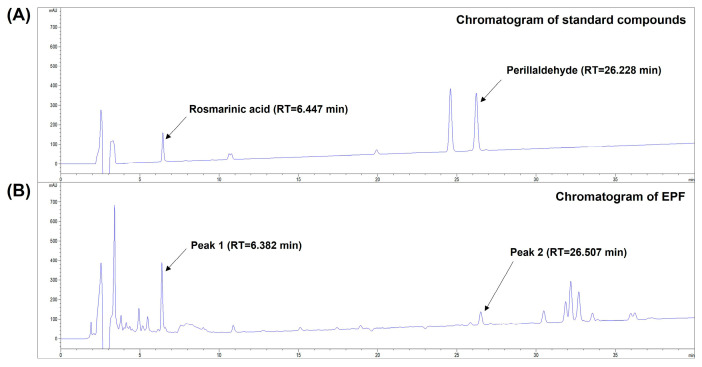
HPLC analyses of standard compounds and EPF. Total HPLC chromatograms of rosmarinic acid, perillaldehyde (**A**), and EPF (**B**) were obtained at a UV wavelength of 233 nm. EPF, ethanol extract of *Perilla frutescens* leaves; HPLC, high-performance liquid chromatography; RT, retention time.

It has been reported that various phytochemical compounds classified as phenolic acids, flavonoids, anthocyanin, volatile oil compounds, and triterpenes are present in PF leaves [19]. HPLC analysis was performed to investigate whether rosmarinic acid, one of the chief phenolic compounds of PF leaves, and perillaldehyde, a predominant volatile compound in PF leaves [19,25], were present in EPF. As shown in Figure 2A,B, the chromatogram of EPF showed multiple peaks, including two peaks at retention times (RTs) of 6.382 min and 26.507 min corresponding to those of rosmarinic acid (RT = 6.447 min) and perillaldehyde (RT = 26.228 min), respectively. These results suggest that rosmarinic acid and perillaldehyde are present in EPF as marker constituents of PF leaves.

### 2.2. Gas Chromatography–Mass Spectrometry (GC-MS) Analysis of EPF

**Figure 3:**
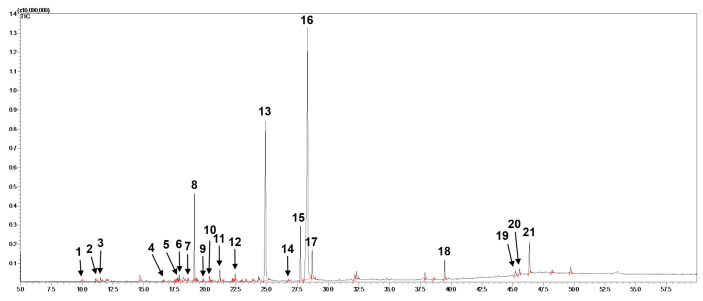
Identification of the constituents in EPF using GC-MS analysis. The total gas chromatogram of EPF is shown. Twenty-one compounds were identified in EPF. EPF, ethanol extract of *Perilla frutescens* leaves; GC-MS, gas chromatography–mass spectrometry.

To identify the constituents of EPF, GC-MS analysis was conducted. Twenty-one compounds classified as volatile compounds, phytosterols, and fatty acids were identified in EPF. Their total chromatogram is shown in Figure 3, and their retention times (RTs), formulae, and molecular weights are described in Table 1. Consistent with our results, previous studies have reported that the majority of these compounds are contained in PF leaves [19].

### 2.3. Determination of a Non-Toxic Concentration Range of EPF in Cancer Cells

**Figure 4:**
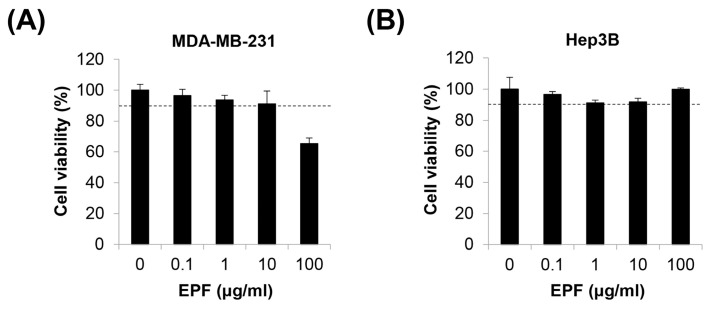
Effects of EPF on the viability of cancer cells. MDA-MB-231 human breast cancer cells (**A**) and Hep3B human hepatocellular carcinoma cells (**B**) were treated with EPF at diverse concentrations (0.1–100 μg/mL) for 24 h. Cell viability was measured by MTT assay. The dotted line indicates 90% cell viability. EPF, ethanol extract of *Perilla frutescens* leaves.

We previously reported that adrenergic agonists can stimulate the migration of MDA-MB-231 human breast cancer (BC) cells and Hep3B human hepatocellular carcinoma (HCC) cells [26]. In this study, we investigated the effects of EPF on adrenergic agonist-induced cancer cell migration. To exclude the possibility that the cytotoxicity of EPF could affect cell migration, we first determined a non-toxic concentration range of EPF in MDA-MB-231 cells and Hep3B cells. We set cell viability ≥ 90% as a non-toxic condition. As shown in Figure 4A,B, cell viability was more than 90% after treatment with 10 μg/mL of EPF in both cell lines, suggesting that EPF ≤10 μg/mL was non-toxic in these cells. EPF showed no cytotoxicity to Hep3B cells, even at 100 μg/mL. However, the same concentration of EPF was toxic to MDA-MB-231 cells, suggesting that the sensitivity to EPF differed according to cell type. Therefore, we determined that the maximum concentration of EPF was 10 μg/mL for MDA-MB-231 cells and 100 μg/mL for Hep3B cells in further experiments.

### 2.4. EPF Suppresses Adrenergic Agonist-Induced Cancer Cell Migration

**Figure 5:**
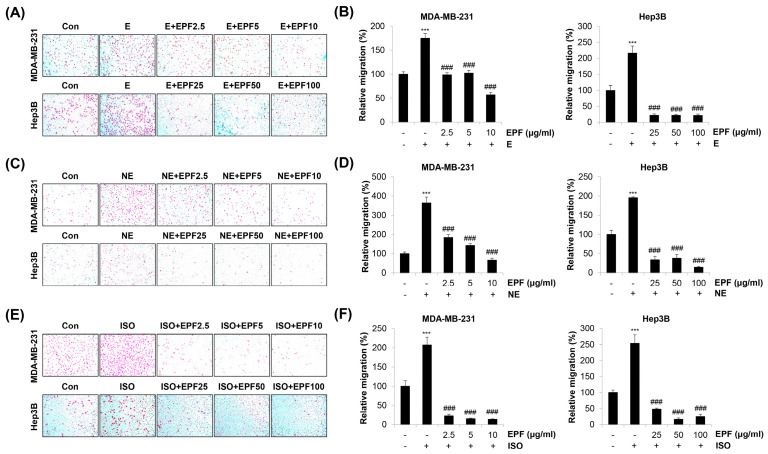
EPF suppresses E/NE/ISO-induced migration of cancer cells. MDA-MB-231 human breast cancer cells and Hep3B human hepatocellular carcinoma cells suspended in serum-free media were seeded into inserts of 24-well Transwell plates and treated with either E (10 μM, **A**,**B**), NE (1 μM, **C**,**D**), or ISO (10 μM, **E**,**F**) for 24 h in the presence of EPF at diverse concentrations (2.5–10 μg/mL for MDA-MB-231; 25–100 μg/mL for Hep3B). Medium containing 10% serum was added to the bottom chambers. After 24 h of incubation, migrated cells were stained and photographed (×100 magnification). Representative images from triplicate analyses are shown (**A**,**C**,**E**). Relative migration compared to that of untreated control cells was evaluated by counting stained cells (**B**,**D**,**F**). The data are expressed as the mean ± SD of three independent experiments. Significance was determined by the Student’s *t*-test (*** *p* < 0.001 vs. untreated controls; ^###^ *p* < 0.001 vs. E/NE/ISO-treated cells). E, epinephrine; NE, norepinephrine; ISO, isoprenaline; EPF, ethanol extract of *Perilla frutescens* leaves.

Our results show that two non-selective adrenergic agonists, E and NE, and a selective β-AR agonist, ISO, commonly increased the migration of MDA-MB-231 cells and Hep3B cells. However, such an increase was significantly abrogated by EPF treatment (Figure 5A–F). As Hep3B cells were treated with relatively higher concentrations (25–100 μg/mL) of EPF compared with MDA-MB-231 cells, we investigated whether the concentration range of EPF applied to MDA-MB-231 cells (2.5–10 μg/mL) exhibited the same anti-migration effect on Hep3B cells. As shown in Supplementary Figure S1A–F, lower concentrations of EPF also suppressed E/NE/ISO-induced migration of Hep3B cells. Taken together, our observations suggest that EPF can inhibit adrenergic agonist-stimulated cancer cell migration.

### 2.5. EPF Inhibits Adrenergic Agonist-Induced Cancer Cell Invasion

**Figure 6:**
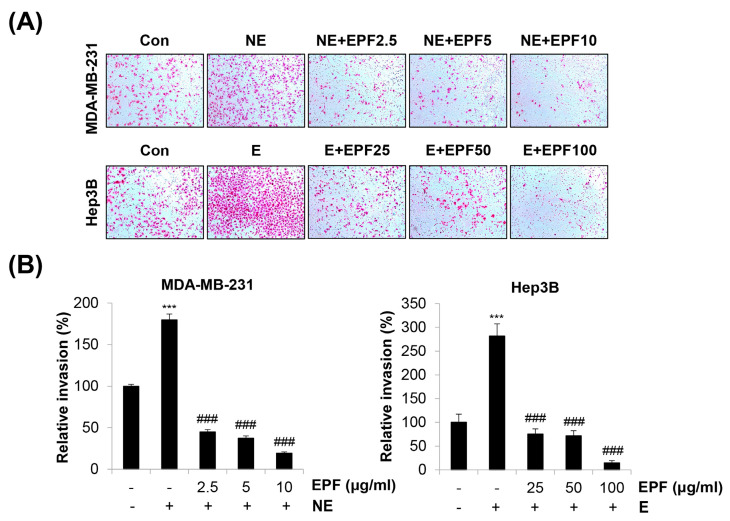
EPF suppresses E/NE-induced invasion of cancer cells. MDA-MB-231 human breast cancer cells (upper panel) and Hep3B human hepatocellular carcinoma cells (lower panel) suspended in serum-free media were seeded into Matrigel-coated inserts of 24-well Transwell plates and treated with either NE (1 μM) or E (10 μM) for 24 h in the presence of EPF at diverse concentrations (2.5-10 μg/mL for MDA-MB-231; 25-100 μg/mL for Hep3B). Medium containing 10% serum was added to the bottom chambers. After 24 h of incubation, invaded cells were stained and photographed (×100 magnification). Representative images from triplicate analyses are shown (**A**). Relative invasion compared to that of untreated control cells was evaluated by counting stained cells (**B**). The data are expressed as the mean ± SD of three independent experiments. Significance was determined by the Student’s *t*-test (*** *p* < 0.001 vs. untreated controls; ^###^ *p* < 0.001 vs. E/NE-treated cells). E, epinephrine; NE, norepinephrine; EPF, ethanol extract of *Perilla frutescens* leaves.

We next investigated the effects of EPF on adrenergic agonist-induced cancer cell invasion. As we previously observed [26], E or NE treatment increased the invasive capacity of MDA-MB-231 cells and Hep3B cells. However, such an increase was completely blocked by EPF treatment (Figure 6A,B). Lower concentrations (2.5–10 μg/mL) of EPF showed similar anti-invasion effects on Hep3B cells (Supplementary Figure S2A,B). These results collectively demonstrate that EPF can suppress adrenergic agonist-stimulated cancer cell invasion.

### 2.6. EPF Regulates EMT Marker Proteins

**Figure 7:**
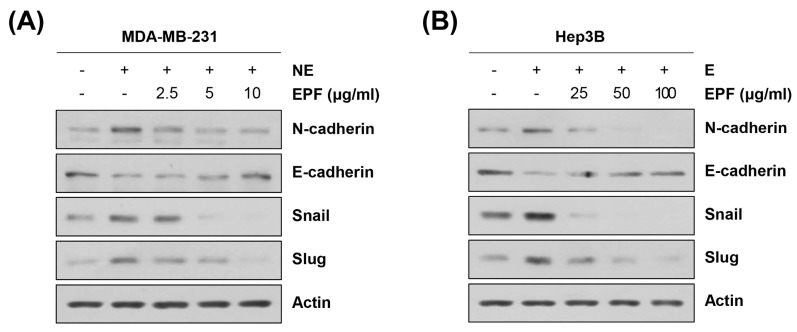
EPF suppresses E/NE-induced EMT in cancer cells. (**A**,**B**) MDA-MB-231 human breast cancer cells (**A**) and Hep3B human hepatocellular carcinoma cells (**B**) were pretreated with EPF at diverse concentrations (2.5–10 μg/mL for MDA-MB-231; 25–100 μg/mL for Hep3B) for 12 h. Cells were then treated with NE (1 μM) or E (10 μM) and incubated for a further 12 h. Expression levels of EMT marker proteins and related transcription factors were determined by Western blot analysis. Actin was used as an internal control. Representative images of duplicate experiments are shown. E, epinephrine; NE, norepinephrine; EPF, ethanol extract of *Perilla frutescens* leaves.

We next determined the mechanism underlying the anti-metastatic effects of EPF. A number of studies have reported that cancer cells can acquire a highly mobile and invasive mesenchymal phenotype to metastasize to other organs [18]. As the β-AR signaling pathway is implicated in the EMT process, we investigated the relationships of antimigration and anti-invasion activities of EPF with EMT regulation [9]. Most studies have suggested that an increase in N-cadherin expression and a decrease in E-cadherin expression are the hallmarks of EMT [18]. N-cadherin is prevalent in non-epithelial tissues, and its expression is correlated with cancer development, as well as angiogenesis [18]. E-cadherin is crucial in maintaining the epithelial phenotype and is downregulated in malignant epithelial cancers [18]. Several transcription factors, including Snail (SNAI1), Slug (SNAI2), ZEB1, and ZEB2, have been reported to suppress the transcription of *E-cadherin* by directly binding to its promoter region [18]. As shown in Figure 7A,B, E or NE treatment increased the expression of N-cadherin but downregulated E-cadherin in MDA-MB-231 cells and Hep3B cells. EPF also upregulated the expression of Snail and Slug, suggesting that adrenergic agonists can induce EMT in cancer cells (Figure 7A,B). EPF treatment dose-dependently reversed the decrease in E-cadherin expression and the increase in N-cadherin, Snail, and Slug expression (Figure 7A,B). These results suggest that EPF can suppress adrenergic agonist-induced metastatic ability in cancer cells by regulating EMT.

### 2.7. Involvement of Src in Regulating EMT and Migration in Cancer Cells

**Figure 8:**
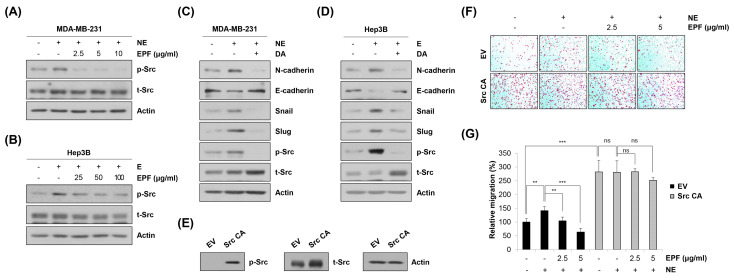
Src mediates the antimetastatic effect of EPF by regulating EMT. (**A**,**B**) MDA-MB-231 human breast cancer cells (**A**) and Hep3B human hepatocellular carcinoma cells (**B**) were pretreated with EPF at diverse concentrations (2.5-10 μg/mL for MDA-MB-231; 25-100 μg/mL for Hep3B) for 22 h. Cells were then treated with NE (1 μM) or E (10 μM) for 2 h to stimulate Src phosphorylation before harvesting. The phosphorylation level and total protein level of Src were determined by Western blot analysis. Representative images of duplicate experiments are shown. (**C**,**D**) MDA-MB-231 cells (**C**) and Hep3B cells (**D**) were pretreated with dasatinib (0.25 μM) for 12 h, followed by NE (1 μM) or E (10 μM) treatment for a further 12 h. Expression levels of EMT marker proteins and related transcription factors were determined by Western blot analysis. Representative images of duplicate experiments are shown. (**E**–**G**) MDA-MB-231 cells were transfected with either empty vector (EV) or constitutively activated Src (Src CA) plasmid. At 48 h post transfection, cells were harvested, and the phosphorylation level and total protein level of Src were determined by Western blot analysis (**E**). At 48 h post transfection, cells were detached and plated into inserts of 24-well Transwell plates and cotreated with NE (1 μM) and EPF (2.5 and 5 μg/mL). As a chemoattractant, 10% FBS medium was used. After 24 h of incubation, migrated cells were stained and photographed (×100 magnification). Representative images from triplicate analyses are shown (**F**). Relative migration compared to that of untreated control cells was evaluated by counting stained cells (**G**). The data are expressed as the mean ± SD of three independent experiments. Significance was determined by the Student’s *t*-test (ns, not significant, ** *p* < 0.01, *** *p* < 0.001 vs. respective control). E, epinephrine; NE, norepinephrine; EPF, ethanol extract of *Perilla frutescens* leaves; DA, dasatinib; EV, empty vector; Src CA, constitutively activated Src (Y527F).

We next determined target molecule(s) of EPF regulating EMT and the metastatic ability of cancer cells. We previously reported that adrenergic agonists can promote migration and invasion of MDA-MB-231 cells and Hep3B cells by activating the β2-AR/Src axis [26], 2022). Therefore, we investigated the role of EPF in regulating Src activity. As shown in Figure 8A,B, E or NE treatment induced Src phosphorylation. However, such induction was reversed by EPF treatment. To determine the involvement of Src in EMT regulation, we next investigated the expression of EMT marker proteins after treatment with dasatinib, a Src kinase inhibitor. Interestingly, E- or NE-induced Src phosphorylation; upregulation of N-cadherin, Snail, and Slug; and downregulation of E-cadherin were significantly reversed by dasatinib treatment in MDA-MB-231 cells and Hep3B cells (Figure 8C,D). These results clearly suggest that adrenergic agonist-stimulated EMT is mediated by Src. To verify whether dephosphorylation of Src by EPF contributed to the anti-metastatic effect of EPF, Src CA plasmid containing constitutively activated Src was used to transfect MDA-MB-231 cells. Strong phosphorylation of Src was detected 48 h post transfection, suggesting that MDA-MB-231 cells were successfully transfected by Src CA plasmid (Figure 8E). In empty vector (EV)-transfected cells, the migratory ability was increased by NE treatment. Such an increase was reversed by cotreatment with NE and EPF. Transfection of Src CA plasmid by itself significantly increased the migration of MDA-MB-231 cells compared to EV-transfected cells. The migratory ability of Src CA-transfected cells was not increased, even after NE treatment, suggesting that activation of Src was enough to mimic the function of NE. In addition, EPF could not suppress the migration of cancer cells any more once Src was hyperactivated, demonstrating that Src was a major target of EPF to exert an antimetastatic effect (Figure 8F,G). Taken together, our observations clearly suggest that EPF can suppress adrenergic agonist-induced cancer cell migration by inhibiting the Src/EMT axis.

## 3. Discussion

In the current study, we investigated inhibitory effects of EPF on the adrenergic agonist-induced metastatic ability of cancer cells and explored the underlying mechanism. PF leaves have traditionally been used to treat stress-related psychological disorders by moving Qi. As chronic stress causes Qi stagnation according to traditional Oriental medicine, moving Qi is a key strategy to manage stress-related symptoms [19,24]. Qi stagnation is also the main mechanism underlying the development and progression of cancer [22,23]. Clinical application of a soothing Qi stagnation method has been suggested for cancer-related depression [27]. Therefore, we postulated that PF leaves could inhibit stress hormone-induced cancer metastasis. Our results clearly show that EPF suppressed E/NE/ISO-induced migration and invasion of MDA-MB-231 cells and Hep3B cells. Blocking of the EMT process by inactivating Src kinase was the main mechanism underlying the antimetastatic effect of EPF. The traditional concept of moving Qi should be interpreted in modern and scientific words. If we understand the molecular mechanism underlying the antimetastatic activity of Qi-moving herbs in a stress-induced metastasis model, the modern meaning of moving Qi can be at least partially clarified. Based on our findings that adrenergic agonists stimulated the metastatic ability of cancer cells via the β2-AR/Src axis [26], which was completely abrogated by EPF treatment, the concept of moving Qi could mean suppression of excessive activation of the β2-AR/Src/EMT axis in chronic stress-induced metastatic cancer. To verify this hypothesis, more studies are needed to investigate the effects of other Qi-moving herbs on cancer metastasis and the β2-AR/Src/EMT axis.

Src is a non-receptor tyrosine kinase known to interact with diverse receptor tyrosine kinases and integrins to activate a variety of downstream targets implicated in cancer cell proliferation, resistance to apoptosis, migration, and invasion [28]. More recently, a novel role of Src in mediating EMT has been suggested. For example, it was found that knockdown of Src or treatment with Src kinase inhibitors downregulated the expression of Slug and vimentin but increased E-cadherin expression in breast cancer cells and head and neck squamous cell carcinoma (HNSCC) cells [29,30]. Highly metastatic MDA-MB-231 cells were found develop an epithelial-like morphology and become more clustered after treatment with an Src inhibitor [29]. Consistently, Lang et al. reported that blocking Src activity with saracatinib can suppress the expression of Snail and vimentin and attenuate the metastatic ability of HNSCC cells [31]. Although how Src regulates EMT in cancer cells remains unclear, previous studies, as well as our current results, collectively suggest that Src can be a promising target to regulate EMT and cancer metastasis.

In future studies, the specific compound exerting antimetastatic effects of EPF should be determined. It has been reported that PF leaves contain a variety of phytochemical compounds [19]. Candidate compounds can be identified by focusing on the following points: (i) Src kinase activity should be inhibited by the candidate compounds, and (ii) candidate compounds should exhibit antimetastatic activity and/or antidepressant effects. It is noteworthy that rosmarinic acid, a representative phenolic compound of PF leaves, can reduce emotional abnormality in mice exposed to conditioned fear stress and decrease the immobility response in a forced swimming test, supporting its antidepressant effect [32,33]. In addition, rosmarinic acid can inhibit metastasis of colorectal cancer and pancreatic cancer by regulating EMT [34,35]. A recent study demonstrated that rosmarinic acid can block Src kinase activity by binding to the active site of c-Src [36]. These previous studies strongly suggest that rosmarinic acid is a probable candidate to mediate antimetastatic activities of EPF. Apigenin, another phenolic-type constituent, can block Src kinase activity and suppress migration and invasion of cancer cells [37,38]. It also exerts an antidepressant effect by regulating dopamine turnover in mouse brains [39]. Perillaldehyde, a predominant volatile compound of PF leaves, can inhibit bone metastasis in prostate cancer with antidepressant activity, although its influence on Src activity has not been reported yet [40,41,42]. Multiple components of EPF can interact in a synergistic fashion to potentiate its antimetastatic activity. An exhaustive literature search, a network pharmacological approach, and social networking among researchers using efficient platforms could help to identify the major constituent(s) of EPF exerting antimetastatic activity in cancer cells [43].

Taken together, the results of the current study demonstrate that EPF can inhibit adrenergic agonist-induced migration and invasion of cancer cells by inactivating the Src/EMT axis. The novel roles of EPF in chronic stress-related cancer metastasis and in EMT regulation were suggested for the first time in this study. Although the specific compound exerting antimetastatic effects of EPF was not determined in this study, our findings suggest that EPF could be used to prevent metastasis in cancer patients, especially those under chronic psychosocial stress. More preclinical and clinical studies are warranted to verify the influence of EPF on chronic stress-related cancer progression.

## 4. Materials and Methods

### 4.1. Preparation of EPF

Dried PF leaves were purchased from Bonchomaru (Seoul, Republic of Korea). PF leaves were collected from Guangdong, China, on 10 March 2022. A voucher sample (No. 062701380) was deposited in the Herbarium of Pathology Laboratory, College of Korean Medicine, Dong-eui University, Busan, Republic of Korea. Dried PF leaves (20 g) were extracted with 80% ethanol (400 mL) in a shaking incubator (100 rpm) at 40 °C for 48 h. Once the first extraction was completed, PF leaves were re-extracted with 100 mL of 80% ethanol for another 24 h at 40 °C. The first extract and the second extract were mixed. The mixture was filtered and concentrated with a vacuum rotary evaporator under reduced pressure. After lyophilization, 3.38 g of EPF powder was obtained. The yield was 16.9%. The powder was reconstituted in dimethyl sulfoxide (DMSO; Amresco, Solon, OH, USA) at 100 mg/mL as a stock solution.

### 4.2. Cell Culture

The MDA-MB-231 human BC cell line and Hep3B human HCC cell line were purchased from American Type Culture Collection (ATCC; Rockville, MD, USA). MDA-MB-231 cells were cultured in RPMI-1640 (WelGENE, Daegu, Korea) supplemented with 100 mL/L of fetal bovine serum (FBS; WelGENE), 100,000 U/L of penicillin (WelGENE), and 100 mg/L of streptomycin (WelGENE). Hep3B cells were grown in Dulbecco’s modified Eagle medium (DMEM, WelGENE) supplemented with 100 mL/L of FBS, 100,000 U/L of penicillin, and 100 mg/L of streptomycin as described above. Both cell lines were grown in a humidified atmosphere under 5% CO_2_ at 37 °C.

### 4.3. HPLC Analysis

Chromatographic analysis was performed with an Agilent 1260 series HPLC system (Agilent Technologies, Palo Alto, CA, USA) using a Shim-pack GIS C18 (250 × 4.6 mm, 5 μm). Standard rosmarinic acid and perillaldehyde were purchased from ChemFaces (Wuhan, China) and dissolved in methanol at 0.1 mg/mL. EPF powder was reconstituted in 70% ethanol at 10 mg/mL. Gradient elution solvents were 0.1% formic acid in water (solvent A) and 0.1% formic acid in acetonitrile (solvent B). The mobile condition was as follows: 70% solvent A and 30% solvent B for 0 min, 25% solvent A and 75% solvent B for 40 min, 70% solvent A and 30% solvent B for 0.5 min, and 70% solvent A and 30% solvent B for 4.5 min. The flow rate was 0.1 mL/min, and the column temperature was 40 °C. The wavelength used for detection was 233 nm.

### 4.4. GC-MS Analysis

For analysis of constituents of EPF, GC-MS analysis was conducted using a GC-MS QP-2010Ultra (Shimadzu, Japan). EPF was dissolved in ethanol at 30 mg/mL. Aliquots (split ratio of 10:1) (1 μL) were then injected into a DB-5MS Ultra column (30 m × 0.25 mm, 0.25 μm). Helium gas was used as the carrier gas at a constant flow rate of 1.0 mL/min. The injection temperature was set at 250 °C. The ion source temperature was 200 °C. The electron energy was 70 eV in electron ionization mode. The oven temperature was programmed from 50 °C (held for 2 min) to 150 °C at 10 °C/min, then to 320 °C at 5 °C/min and held at the final temperature for 14 min. The scan interval was 0.3 s, and the fragment size ranged from 40 to 500 m/z. Total GC detection was completed in 60 min.

### 4.5. MTT Assay

First, 3 × 10^3^ cells/well were seeded into 96-well plates and stabilized overnight. These cells were treated with EPF at different concentrations (0.1–100 μg/mL) and incubated at 37 °C for 24 h. At 24 h post treatment, MTT solution (3-(4,5-dimethylthiazol-2-yl)-2,5-diphenyltetrazolium bromide; Duchefa, Haarlem, The Netherlands) was added to the culture medium at 0.4 mg/mL. After 2 h of incubation at 37 °C, the culture medium was discarded, and 100 μL DMSO was added into each well to thoroughly dissolve MTT formazan. Cell viability was evaluated by measuring the absorbance of each well at 540 nm using a microplate reader (SpectraMax M3; Molecular Devices, San Jose, CA, USA).

### 4.6. Transwell Assay

The migration ability of cancer cells was measured with a Transwell migration assay. Briefly, 2 × 10^4^ cells (Hep3B) or 3 × 10^4^ cells (MDA-MB-231) were suspended in 200 μL serum-free medium and seeded onto inserts of a 24-well Transwell plate with an 8 μm pore size (Corning Costar, Lowell, MA, USA), outer membrane of which was coated with 0.1% gelatin (Sciencell, Carlsbad, CA, USA). Cells were then treated with EPF at different concentrations (0.1–100 μg/mL) in the presence of different kinds of adrenergic agonists including E, NE, and ISO (Sigma-Aldrich, St. Louis, MO, USA). Medium containing 10% FBS (500 μL) was added to the bottom chambers of a 24-well Transwell plate as a chemoattractant. After 24 h of incubation, Transwell membranes were fixed with methanol for 5 min, stained with hematoxylin (Sigma-Aldrich) for 30 min at room temperature, and washed several times with distilled water (DW). Immediately before image acquisition, dried membranes were separated from inserts and mounted onto glass slides. Stained cells were considered migrated cells and photographed under a microscope (Leica, Wetzlar, Germany) at 100× magnification. The invasive property of cancer cells was evaluated by coating the inner membranes of inserts with 300 μg/mL Matrigel (BD Bioscience, San Jose, CA, USA). Other steps of the Transwell invasion assay were the same as the Transwell migration assay described above.

### 4.7. Western Blot Analysis

The methodology of Western blot analysis was previously described in detail [44]. Briefly, total proteins were extracted using cold RIPA buffer (Thermo Fisher Scientific, Waltham, MA, USA). After protein quantification using a bicinchoninic acid (BCA) protein assay kit (Pierce Biotechnology, Rockford, IL, USA), proteins (20 µg) were separated by sodium dodecyl sulfate-polyacrylamide gel electrophoresis (SDS-PAGE) and transferred onto polyvinyl difluoride (PVDF) membranes (Millipore, Bedford, MA, USA). Membranes were then blocked with 3% bovine serum albumin (BSA, GenDEPOT, Katy, TX, USA) and probed with corresponding primary antibodies (1:1000 dilution) and secondary antibodies (1:5000 dilution). The expression-level of target protein was detected using a D-Plus ECL Femto system (Donginbio, Seoul, Korea). Primary antibodies against Snail (#3879S), Slug (#9585S), N-cadherin (#13116S), E-cadherin (#3195S), and phospho-Src (Y416, #2101S) were purchased from Cell Signaling Technology (Beverly, MA, USA), and those against Src (#sc-8056) and actin (#sc-47778) were purchased from Santa Cruz Biotechnology (Santa Cruz, CA, USA). An anti-mouse IgG secondary antibody was purchased from Bethyl Laboratories (Montgomery, TX, USA), and an anti-rabbit IgG secondary antibody was purchased from Enzo Life Sciences (Farmingdale, NY, USA).

### 4.8. Transfection

A constitutively active Src plasmid (pcSrc527, #17675) and an empty vector (pEVX, #17666) were purchased from Addgene. After 3 × 10^5^ cells were seeded into 6-well plates, cells were transfected with 1 µg pcSrc527 or pEVX using Lipofectamine 2000 (Invitrogen, Carlsbad, CA, USA). At 48 h post transfection, cells were collected and subjected to Western blot analysis to determine transfection efficiency or seeded (3 × 10^4^ cells/well) into inserts of 24-well Transwell plates and cotreated with NE (1 µM) and EPF (2.5–5 µM). After 24 h of incubation, migrated cells were stained and photographed as described above.

### 4.9. Statistical Analysis

Data are presented as mean ± standard deviation (SD). Statistical comparisons between two groups were performed with a paired Student’s *t*-test, and *p* < 0.05 was considered statistically significant.

## Figures and Tables

**Table 1:** Characterization of chemical constituents in EPF by GC-MS analysis.

Peak # ^1^	RT ^2^ (min)	Formula	MW ^3^ (m/z)	Identification
1	9.937	C_6_H_8_O_4_	144	2,3-Dihydro-3,5-dihydroxy-6-methyl-4H-pyran-4-one
2	11.118	C_8_H_8_O	120	2,3-Dihydro-benzofuran
3	11.473	C_5_H_10_O_4_	134	2,3-Dihydroxypropyl acetate
4	16.569	C_11_H_16_O_2_	180	Dihydroactinidiolide
5	17.508	C_15_H_24_O	220	(-)-Spathulenol
6	17.647	C_15_H_24_O	220	(-)-Caryophyllene oxide
7	18.595	C_12_H_16_O_3_	208	Isoelemicin
8	19.125	C_12_H_16_O_3_	208	cis-Asarone
9	19.833	C_10_H_12_O_4_	196	Benzaldehyde, 2,4,5-trimethoxy-
10	20.335	C_12_H_16_O_4_	224	1-(2,4,5-Trimethoxyphenyl)propan-2-one
11	21.183	C_12_H_14_O_4_	222	Isodillapiole
12	22.441	C_20_H_38_	278	Neophytadiene
13	24.919	C_16_H_32_O_2_	256	Palmitic acid
14	26.764	C_17_H_34_O_2_	270	Heptadecanoic acid
15	27.748	C_20_H_40_O	296	Phytol
16	28.338	C_18_H_30_O_2_	278	Linolenic acid
17	28.703	C_18_H_36_O_2_	284	stearic acid
18	39.469	C_30_H_50_	410	Squalene
19	45.213	C_28_H_48_O	400	Campesterol
20	45.551	C_29_H_48_O	412	stigmasterol
21	46.355	C_29_H_50_O	414	Clionasterol

^1^ #, number; ^2^ RT, retention time; ^3^ MW, molecular weight.

## Data Availability

Data supporting the findings of this study are available from the corresponding author upon reasonable request.

## Supplementary Materials

The following supporting information can be downloaded at: https://www.mdpi.com/article/10.3390/molecules28083414/s1, Figure S1: Effects of low concentrations of EPF on E/NE/ISO-induced migration of Hep3B cells.; Figure S2: Effects of low concentrations of EPF on E-induced invasion of Hep3B cells.

## References

[1] Oh H.M., Son C.G. (2021). The Risk of Psychological Stress on Cancer Recurrence: A Systematic Review. Cancers.

[2] Batty G.D., Russ T.C., Stamatakis E., Kivimäki M. (2017). Psychological distress in relation to site specific cancer mortality: Pooling of unpublished data from 16 prospective cohort studies. BMJ.

[3] Kim-Fuchs C., Le C.P., Pimentel M.A., Shackleford D., Ferrari D., Angst E., Hollande F., Sloan E.K. (2014). Chronic stress accelerates pancreatic cancer growth and invasion: A critical role for beta-adrenergic signaling in the pancreatic microenvironment. Brain Behav. Immun..

[4] Thaker P.H., Han L.Y., Kamat A.A., Arevalo J.M., Takahashi R., Lu C., Jennings N.B., Armaiz-Pena G., Bankson J.A., Ravoori M. (2006). Chronic stress promotes tumor growth and angiogenesis in a mouse model of ovarian carcinoma. Nat. Med..

[5] Chang A., Le C.P., Walker A.K., Creed S.J., Pon C.K., Albold S., Carroll D., Halls M.L., Lane J.R., Riedel B. (2016). β2-Adrenoceptors on tumor cells play a critical role in stress-enhanced metastasis in a mouse model of breast cancer. Brain Behav. Immun..

[6] Gudenkauf L.M., Ehlers S.L. (2018). Psychosocial interventions in breast cancer survivorship care. Breast.

[7] Fawzy F.I., Fawzy N.W., Hyun C.S., Elashoff R., Guthrie D., Fahey J.L., Morton D.L. (1993). Malignant melanoma. Effects of an early structured psychiatric intervention, coping, and affective state on recurrence and survival 6 years later. Arch. Gen. Psychiatry.

[8] Reiche E.M., Nunes S.O., Morimoto H.K. (2004). Stress, depression, the immune system, and cancer. Lancet Oncol..

[9] Cole S.W., Sood A.K. (2012). Molecular pathways: Beta-adrenergic signaling in cancer. Clin. Cancer Res..

[10] Abrass C.K., O’Connor S.W., Scarpace P.J., Abrass I.B. (1985). Characterization of the beta-adrenergic receptor of the rat peritoneal macrophage. J. Immunol..

[11] Graf K., Gräfe M., Dümmler U., O’Connor A., Regitz-Zagrosek V., Kunkel G., Auch-Schwelk W., Fleck E. (1993). Regulation of beta-adrenergic receptors on endothelial cells in culture. Eur. Heart J..

[12] Melhem-Bertrandt A., Chavez-Macgregor M., Lei X., Brown E.N., Lee R.T., Meric-Bernstam F., Sood A.K., Conzen S.D., Hortobagyi G.N., Gonzalez-Angulo A.M. (2011). Beta-blocker use is associated with improved relapse-free survival in patients with triple-negative breast cancer. J. Clin. Oncol..

[13] Parada-Huerta E., Alvarez-Dominguez T., Uribe-Escamilla R., Rodriguez-Joya J., Ponce-Medrano J.D., Padron-Lucio S., Alfaro-Rodriguez A., Bandala C. (2016). Metastasis Risk Reduction Related with Beta-Blocker Treatment in Mexican Women with Breast Cancer. Asian Pac. J. Cancer Prev..

[14] Udumyan R., Montgomery S., Fang F., Almroth H., Valdimarsdottir U., Ekbom A., Smedby K.E., Fall K. (2017). Beta-Blocker Drug Use and Survival among Patients with Pancreatic Adenocarcinoma. Cancer Res..

[15] Nkontchou G., Aout M., Mahmoudi A., Roulot D., Bourcier V., Grando-Lemaire V., Ganne-Carrie N., Trinchet J.C., Vicaut E., Beaugrand M. (2012). Effect of long-term propranolol treatment on hepatocellular carcinoma incidence in patients with HCV-associated cirrhosis. Cancer Prev. Res..

[16] Chang H., Lee S.H. (2022). Beta-adrenergic receptor blockers and hepatocellular carcinoma survival: A systemic review and meta-analysis. Clin. Exp. Med..

[17] Chambers A.F., Groom A.C., MacDonald I.C. (2002). Dissemination and growth of cancer cells in metastatic sites. Nat. Rev. Cancer.

[18] Micalizzi D.S., Farabaugh S.M., Ford H.L. (2010). Epithelial-mesenchymal transition in cancer: Parallels between normal development and tumor progression. J. Mammary Gland Biol. Neoplasia.

[19] Ahmed H.M. (2018). Ethnomedicinal, Phytochemical and Pharmacological Investigations of *Perilla frutescens* (L.) Britt. Molecules.

[20] Kim C.L., Shin Y.S., Choi S.H., Oh S., Kim K., Jeong H.S., Mo J.S. (2021). Extracts of *Perilla frutescens* var. *Acuta* (Odash.) Kudo Leaves Have Antitumor Effects on Breast Cancer Cells by Suppressing YAP Activity. Evid. Based Complement. Alternat. Med..

[21] Maeda A., Fujimura T., Hirakawa N., Baba K., Kawamoto S. (2022). A Methoxyflavanone from Perilla frutescens Induces Cellular Senescence in A549 Human Lung Adenocarcinoma Cells but Not in Normal Human Bronchial Epithelial Cells. Biol. Pharm. Bull..

[22] Zhou X., Tian D. (2001). A review on nasopharyngeal carcinoma in ancient Chinese literature. Zhonghua Yi Shi Za Zhi.

[23] Zhang Y.H., Qin X., Xu J. (2012). Analysis of Chinese medical syndrome features of patients with primary liver cancer before and after transcatheter arterial chemoembolization. Zhongguo Zhong Xi Yi Jie He Za Zhi.

[24] Huang H., Song Q., Chen J., Zeng Y., Wang W., Jiao B., Lin J., Li Y., Zhang R., Ma L. (2022). The Role of Qi-Stagnation Constitution and Emotion Regulation in the Association Between Childhood Maltreatment and Depression in Chinese College Students. Front. Psychiatry.

[25] Liu J., Wan Y., Zhao Z., Chen H. (2013). Determination of the content of rosmarinic acid by HPLC and analytical comparison of volatile constituents by GC-MS in different parts of *Perilla frutescens* (L.) Britt. Chem. Cent. J..

[26] Jeong J.H., Park H.J., Park S.H., Choi Y.H., Chi G.Y. (2022). β2-Adrenergic Receptor Signaling Pathway Stimulates the Migration and Invasion of Cancer Cells via Src Activation. Molecules.

[27] Zhang J., Liu Y., Xu Y. (2020). Soothing liver-qi stagnation method for cancer-related depression: A protocol for systematic review and meta-analysis. Medicine.

[28] Zhang S., Yu D. (2012). Targeting Src family kinases in anti-cancer therapies: Turning promise into triumph. Trends Pharmacol. Sci..

[29] Liu X., Feng R. (2010). Inhibition of epithelial to mesenchymal transition in metastatic breast carcinoma cells by c-Src suppression. Acta Biochim. Biophys. Sin..

[30] Srivastava K., Pickard A., Craig S.G., Quinn G.P., Lambe S.M., James J.A., McDade S.S., McCance D.J. (2018). ΔNp63γ/SRC/Slug Signaling Axis Promotes Epithelial-to-Mesenchymal Transition in Squamous Cancers. Clin. Cancer Res..

[31] Lang L., Shay C., Xiong Y., Thakkar P., Chemmalakuzhy R., Wang X., Teng Y. (2018). Combating head and neck cancer metastases by targeting Src using multifunctional nanoparticle-based saracatinib. J. Hematol. Oncol..

[32] Takeda H., Tsuji M., Inazu M., Egashira T., Matsumiya T. (2002). Rosmarinic acid and caffeic acid produce antidepressive-like effect in the forced swimming test in mice. Eur. J. Pharmacol..

[33] Takeda H., Tsuji M., Miyamoto J., Matsumiya T. (2002). Rosmarinic acid and caffeic acid reduce the defensive freezing behavior of mice exposed to conditioned fear stress. Psychopharmacology.

[34] Han Y.H., Kee J.Y., Hong S.H. (2018). Rosm arinic Acid Activates AMPK to Inhibit Metastasis of Colorectal Cancer. Front. Pharmacol..

[35] Han Y., Ma L., Zhao L., Feng W., Zheng X. (2019). Rosmarinic inhibits cell proliferation, invasion and migration via up-regulating miR-506 and suppressing MMP2/16 expression in pancreatic cancer. Biomed. Pharmacother..

[36] Highland H.N., Thakur M.B., George L.B. (2022). Controlling non-small cell lung cancer progression by blocking focal adhesion kinase-c-Src active site with *Rosmarinus officinalis* L. phytocomponents: An in silico and in vitro study. J. Cancer Res. Ther..

[37] Byun S., Park J., Lee E., Lim S., Yu J.G., Lee S.J., Chen H., Dong Z., Lee K.W., Lee H.J. (2013). Src kinase is a direct target of apigenin against UVB-induced skin inflammation. Carcinogenesis.

[38] Rahmani A.H., Alsahli M.A., Almatroudi A., Almogbel M.A., Khan A.A., Anwar S., Almatroodi S.A. (2022). The Potential Role of Apigenin in Cancer Prevention and Treatment. Molecules.

[39] Nakazawa T., Yasuda T., Ueda J., Ohsawa K. (2003). Antidepressant-like effects of apigenin and 2,4,5-trimethoxycinnamic acid from Perilla frutescens in the forced swimming test. Biol. Pharm. Bull..

[40] Ji W.W., Li R.P., Li M., Wang S.Y., Zhang X., Niu X.X., Li W., Yan L., Wang Y., Fu Q. (2014). Antidepressant-like effect of essential oil of Perilla frutescens in a chronic, unpredictable, mild stress-induced depression model mice. Chin. J. Nat. Med..

[41] Lin Z., Huang S., LingHu X., Wang Y., Wang B., Zhong S., Xie S., Xu X., Yu A., Nagai A. (2022). Perillaldehyde inhibits bone metastasis and receptor activator of nuclear factor-κB ligand (RANKL) signaling-induced osteoclastogenesis in prostate cancer cell lines. Bioengineered.

[42] Erhunmwunsee F., Pan C., Yang K., Li Y., Liu M., Tian J. (2022). Recent development in biological activities and safety concerns of perillaldehyde from perilla plants: A review. Crit. Rev. Food Sci. Nutr..

[43] Singla R.K., De R., Efferth T., Mezzetti B., Sahab Uddin M., Sanusi, Ntie-Kang F., Wang D., Schultz F., Kharat K.R. (2023). The International Natural Product Sciences Taskforce (INPST) and the power of Twitter networking exemplified through #INPST hashtag analysis. Phytomedicine.

[44] Min T.R., Park H.J., Ha K.T., Chi G.Y., Choi Y.H., Park S.H. (2019). Suppression of EGFR/STAT3 activity by lupeol contributes to the induction of the apoptosis of human non-small cell lung cancer cells. Int. J. Oncol..

