# Supplementary material for: An Ethanol Extract of Perilla frutescens Leaves Suppresses Adrenergic Agonist-Induced Metastatic Ability of Cancer Cells by Inhibiting Src-Mediated EMT
Source: Molecules. 2023 Apr 12;28(8):3414. doi: 10.3390/molecules28083414 (PMC10141214; doi:10.3390/molecules28083414)
Supplement: Supplementary file 1 [file molecules-28-03414-s001.zip › molecules-2304373-supplementary.pdf]

Supplementary Material

An Ethanol Extract of Perilla frutescens Leaves Suppresses Adrenergic Agonist-Induced Metastatic Ability of Cancer Cells by Inhibiting Src-Mediated EMT

Jae-Hoon Jeong 1,†, Hyun-Ji Park 1,†, Gyoo-Yong Chi 1, Yung-Hyun Choi 2,* and Shin-Hyung Park 1,*

1 Department of Pathology, College of KoreanMedicine, Dong-eui University, Busan 47227, Republic of Korea; 15224@deu.ac.kr (J.-H.J.); 14554@deu.ac.kr (H.-J.P.); cgyu@deu.ac.kr (G.-Y.C.)
2 Department of Biochemistry, College of Korean Medicine, Dong-eui University, Busan 47227, Republic of Korea
* Correspondence: choiyh@deu.ac.kr (Y.-H.C.); omdpark@deu.ac.kr (S.-H.P.); Tel.: +82-51-890-3319 (Y.-H.C.); +82-51-890-3332 (S.-H.P.)
† These authors contributed equally to this work.

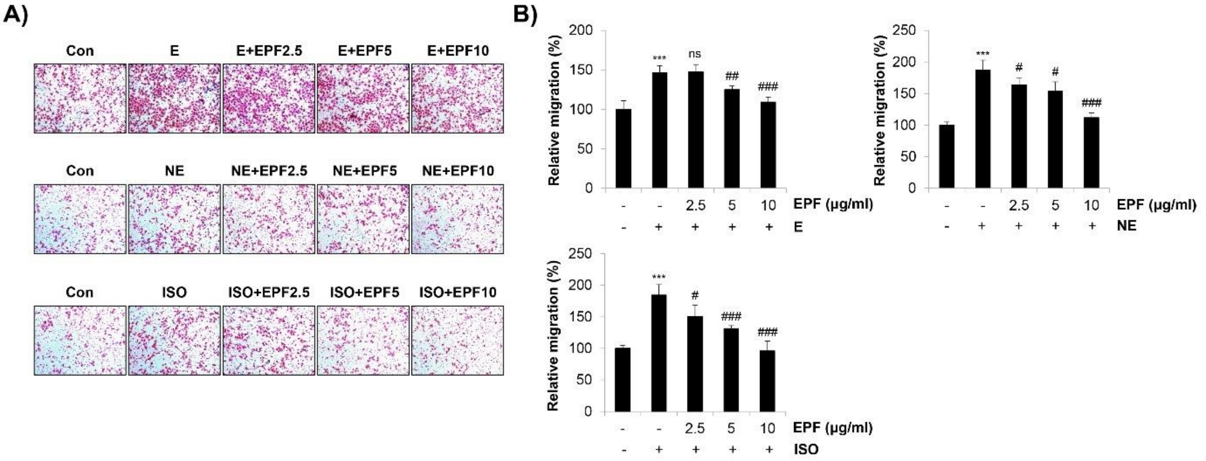

Fugure S1. Effects of low concentrations of EPF on E/NE/ISO-induced migration of Hep3B cells. Hep3B human hepatocellular carcinoma cells suspended in serum free media were seeded into inserts of 24-well transwell plates and treated with E (10 μM), NE (1 μM), or ISO (10 μM) for 24 h in the presence of EPF at diverse concentrations (2.5–10 μg/mL). Bottom chambers were filled with 10% FBS media. After 24 h of incubation, migrated cells were stained and photographed (×100 magnification). Representative images from triplicate analyses are shown (A). Relative migration compared to that of untreated control cells was evaluated by counting stained cells (B). The data are expressed as the mean ± SD of three independent experiments. Significance was determined by the Student's t-test (*** $p < 0.001$ vs. untreated controls; ns, not significant, $^{\#}$ $p < 0.05$, $^{\#\#}$ $p < 0.01$, $^{\#\#\#}$ $p < 0.001$ vs. E/NE/ISO-treated cells). E, epinephrine; NE, norepinephrine; ISO, isoprenaline; EPF, ethanol extract of *Perilla frutescens* leaves.

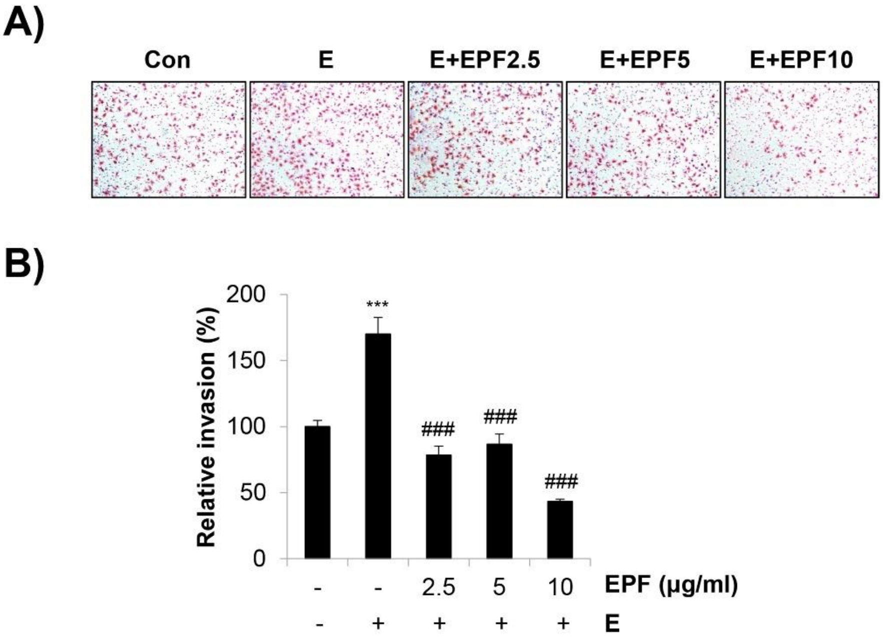

Figure S2. Effects of low concentrations of EPF on E-induced invasion of Hep3B cells. Hep3B human hepatocellular carcinoma cells suspended in serum free media were seeded into Matrigel-coated inserts of 24-well transwell plates and treated with E (10 μM) for 24 h in the presence of EPF at diverse concentrations (2.5–10 μg/mL). Bottom chambers were filled with 10% FBS media. After 24 h of incubation, invaded cells were stained and photographed (×100 magnification). Representative images from triplicate analyses are shown (A). Relative invasion compared to that of untreated control cells was evaluated by counting stained cells (B). The data are expressed as the mean ± SD of three independent experiments. Significance was determined by the Student's t-test (*** $p < 0.001$ vs. untreated controls; $^{\#\#\#}$ $p < 0.001$ vs. E-treated cells). E, epinephrine; EPF, ethanol extract of *Perilla frutescens* leaves.
